# Patterns, factors associated and morbidity burden of asthma in India

**DOI:** 10.1371/journal.pone.0185938

**Published:** 2017-10-26

**Authors:** Prakash Kumar, Usha Ram

**Affiliations:** 1 International Institute for Population Sciences, Mumbai, Maharashtra, India; 2 Department of Public Health and Mortality Studies, International Institute for Population Sciences, Mumbai, Maharashtra, India; St. Michael's Hospital, CANADA

## Abstract

**Background:**

Asthma is a non-curable but preventable disease, responsible for higher morbidity worldwide. According to recent WHO report, nearly 235 million people are suffering from asthma leading to 383000 deaths in 2015. The burden of asthma morbidity is higher in developed countries and is increasing in developing countries.

**Objective:**

The present study was aimed at studying the change in prevalence rate of asthma, associated risk factors and estimation of morbidity burden and avoidable cases of asthma in India.

**Methods:**

The second round of Indian Human Development Survey (IHDS-II), 2011–12, was used for the study. For the present study, asthma was defines as ever diagnosed with asthma or having cough with short breath. Multiple-logistic regression was used to identify the possible risk factors associated with prevalence of reporting asthma. Population attributable fractions (PAFs) were computed to estimate the overall and risk factors specific burden of morbidity due to asthma using the extrapolated population of year 2015 using 2011 census.

**Results:**

Overall prevalence rate of asthma increased from 41.9 (per 1000 population) in 2004–05 to 54.9 (per 1000 population) in 2011–12. The prevalence rate of reporting asthma was higher in poorer states compared to richer states, and also varied by sub-geographies, with higher prevalence rate in northern states of the country and lower rates in north-eastern states of the country. The odds of reporting asthma was higher for younger and older ages, individual with fewer years of schooling (OR: 1.41; 95% CI: 1.21–1.64) for individual with zero years of schooling compared to those with 11 or more years of schooling, individual from lower economic status, individual living in household using unclean fuels (OR:1.21; 95% CI: 1.08–1.34) and smokers (OR: 1.34; 95% CI: 1.17–1.55) compared to their counterparts. In the year 2015, the overall morbidity burden of asthma was estimated at nearly 65 million and more than 82 thousand deaths were attributed due to asthma. The burden was highest among individuals living in households using solid fuels (firewood~80%, Kerosene~78%). One-third of the cases could be eliminated by minimising the use of any solid fuels. Around 17% of all the asthma cases in population could be attributed to underweight.

**Conclusion:**

Eliminating the modifiable risk factors could help reduce in huge amount of asthma cases for example by providing education, cessation in smoking, and schemes like *Pradhan Mantri Ujjwala Yojana* (PMUY), by providing clean fuel (LPG) to poor and vulnerable households.

## Introduction

The Director General at the 61^st^ World Health Assembly, 2008 stated that heart diseases and cancers are the leading killers whereas Diabetes and Asthma are on the rise [1]. Chronic respiratory diseases account for 4.7% of global DALYs, out of which asthma making up a fifth of the total [2]. According to Global Asthma Report (2014), asthma affects approximately 334 million people worldwide and responsible for 345,736 deaths annually (1 in 150 deaths worldwide) [3]. Same reports also indicated that asthma is the 14^th^ most important disease disorder as far as extent and duration of disability is considered. Around 14% of world’s children and 8.6% of young adults had experienced asthma symptoms. Asthma is a preventable chronic disease, caused by resistance in airflow in the airways of lungs. Appropriate management and proper medical treatment of Asthma [3] can prevent most of the asthma related deaths, yet, worldwide, one out of every 250 deaths is attributed to Asthma. Global prevalence rate of doctor diagnosed asthma, clinical/treated asthma and wheezing in adults were 4.3%, 4.5% and 8.6% respectively [4]. Further they found that the prevalence rate of asthma varies widely across countries; from a high of 21% for Australia to a low of 0.2% for China. Not only do prevalence rates of asthma show wide variations across countries and regions, the rates indicate an upward trend over time [3], primarily in the middle and low income countries between 1993 and 2003. By year 2025, additional 100 million more cases of asthma are expected globally [5]. Asthma poses greater public health challenges for most countries regardless of their economic status. Although, prevalence is observed to be high in developed countries, it is more fatal in the developing countries accounting for nearly 80% of asthma deaths worldwide [6].

Asthma emerged as the 25^th^ leading causes of years of life lost (YLLs) due to premature mortality in 2010 accounting for around one percent of total YLLs due to premature mortality [7]. Compared to the average prevalence of asthma in Asia (3.5%), India reported slightly lower rate at 3 percent [3]. There are a few studies that have explored issues related to asthma in India [6–8]. Further, most of these studies examined the issue only for a smaller area excepting few that are multi-centric in nature. The Indian Study on Epidemiology of Asthma, Respiratory Symptoms and Chronic Bronchitis (INSEARCH) among adults using a validated International Union against Tuberculosis and Lung Diseases questionnaire [6, 8] estimated the prevalence rate of asthma at 2.05% between years 2007–09 with an estimated burden of about 17.23 million by year 2011. Agrawal, Pearce, and Ebrahim (2013) estimated the prevalence rate of self-reported asthma at around 2% using data from National Family Health Survey– 3 conducted during year 2005–06 [6]. Agnihotram and Chattopadyay (2005) found strong association between respiratory disorders and poverty and unhealthy environment. They also noted wide variation across country with higher prevalence rates in Karnataka, Gujarat, Haryana, Uttar Pradesh, Kerala and Madhya Pradesh and attributed asthma as the leading cause of death in rural India [9]. Asthmatic persons are more likely to report poor self-rated health, more days of impaired physical days each month of impaired physical or mental health, almost double that of those who never have had asthma [10].

There is a lack of understanding on the prevalence rate of asthma and local variations in it in the recent times, especially post 2003. Little is known about the risk factors associated with asthma. In the present paper we attempt to address some of these questions for India and its region using most recent data available. The analysis specifically examines whether there is an increase in the prevalence rate of asthma in India and its sub-regions? Does prevalence of asthma indicate any regional variation? What are the prominent socioeconomic, demographic and life-style risk factors associated with asthma? And, finally, how many asthma cases could be avoided/prevented by intervening the modifiable risk factors addressed?

## Data source and methods

The study uses data from the second round of Indian Human Development Survey (IHDS) conducted in 2011–12. The IHDS is a nationally representative, multi-topic panel survey and covers all states and the union territories of India except Andaman and Nicobar Islands and Lakshadweep. It surveyed a total of 42,152 households from 1,503 villages and 971 urban localities. The total surveyed population was 204,568 individual, for which data was collected and used in the present study for analysis.

The survey collected information on various aspects including health, education, employment, economic status, marriage, fertility, gender relations, and social capital. Information regarding the socio-economic conditions of the households was collected from head of households and information regarding health, education, fertility, family planning, marriage and gender relations in the household and community was collected from the ever-married women aged 15–49 years. Height and weight measurement of children under age 5, aged 8–11, and their mothers were also collected. For the present study, household and individual data files were used. The household file contains data on household characteristics such as type of house, fuel used, religion, *chulha* type (cooking stove) etc. The individual file contains information on individual characteristics including age, sex, education, occupation, religion, caste, etc. The extracted information from both household and individual files was merged and analyzed using STATA 13. To calculate the national burden of asthma morbidity, the standardized prevalence rate estimates for men and women across different age groups were summed and multiplied by the extrapolated number of individuals in each stratum using Census of India 2011 numbers.

The prevalence rate in the study was defined as persons suffering/reporting from asthma per 1000 population. Group comparison was performed using Chi-square test to examine the statistical significance of association of asthma and various socio-economic-demographic, and life-style characteristics. Principle component analysis (PCA) was used to construct the wealth quintile using land and house ownership and household assets. Linear logistic regression [11]was used to examine the relationship of selected socio-economic-demographic and life-style factors with asthma. Further, following four models using multi-variable linear logistic regression were identified.

1. Model 1: includes demographic variables only
2. Model 2: includes life-style variables only
3. Model 3: includes socio-economic variables only
4. Model 4: Combine model including all the socio-economic-demographic and life-style variables.

In the survey, weight and height of all individual were not collected, it was only measured for children under age-5, 8–11 years, and their mothers. But the morbidity condition was recorded for all the household members. Hence, multiple imputation technique using logit regression model was used to impute the body mass index (BMI) for missing values considering socio-economic variable like age, sex, place of residence, religion, caste and wealth quintile as background variable.

To estimate the morbidity burden of asthma due to various modifiable risk factors, Population Attributable Fractions (PAFs) were calculated and further total number of avoidable cases were estimated for the year 2015. The population of India for 2015 was obtained by extrapolating census 2011 population distribution. First, total population for year 2015 was estimated considering the population annual growth rate between 2001 and 2011 census. Then the gender specific total population was estimated using the annual growth rate from 2001 to 2011 and was imposed on the age distribution of 2011 to get the 2015 gender specific age distribution. Population attributable fraction (PAF) is the percentage of disease in the entire population that can be attributed to the exposure [12]. In other words, if the whole population is considered at the same risk of disease as the individuals who either had little or no exposure for the same environment, then the proportion by which prevalence reduces (or incidence) of the disease (in our case asthma) can be defined as PAF.

Symbolically,

$$PAF={Proportion}_{diseased}*AP=\frac{\left[{PE}_{tp}*(RR-1)\right]}{\left[{PE}_{tp}*(RR-1)+1\right]}$$

Where,

*RR =* Relative risk ratio; defined as prevalence rate among exposed divided by prevalence rate among unexposed
*AP* = Attributable proportion (or fraction); usually gives percentage of disease in the exposed group that can be attributed to the exposure
*PE*_*tp*_ = Proportion of population exposed out of the total population of the area

The variables used in the analysis are categorized as below:

### Dependent variable

The dependent variable used in the study is members suffering from Asthma. In the survey household head was asked to report if any usual member of the household was ever diagnosed with asthma. Additionally, the head was also asked to report if any usual member suffered from cough with short breath during the past 30 days prior to the survey date. In view of this, for the present study, we defined asthma cases based on two criteria, that is, if a member was either diagnosed with asthma or suffered with short breath. Thus, the dependent variable used was dichotomous classified as diagnosed with asthma or having cough with short breath = ‘1’ and ‘0’ otherwise.

### Independent variables

1. Demographic characteristics: It includes member age (below-5 years, 5–14 years, 15–29 years, 30–44 years, 45–64 years, and 65 years and above),sex, marital status (currently married, never married, others), completed years of education (0 years including those who never attended school, 1–5 years, 6–10 years and 11 years and above).
2. Socio-economic characteristics: It includes religion categorized into three category (Hindu, Muslim, Others), Caste category (General, other backward classes, scheduled castes, scheduled tribe), place of residence (rural, urban), wealth quintile divided five quintile (poorest, poor, middle, rich, richest), type of fuel used (clean only, others) and hours burning stove (dichotomous for bivariate analysis with cutoff limit of 3 hours whereas continuous for regression analysis), Type of house classified in three-categories (*pakka* houses: made from high-quality materials such as bricks, tiles, cement and concrete throughout; *kachha* houses: made from mud, thatch or other low-quality materials; *semi-pakka* houses: made from partly low-quality materials and partly high-quality materials).
3. Life-style characteristics: It includes variables such as, ever smoked either cigaretteor *biddi*,(Yes, No) chewing tobacco behavior (Yes, No), consumption of alcohol (Yes No), and nutritional status was captured using body mass index (Underweight: BMI<18.5 Kg/m^2^; Normal weight: BMI: 18.5–24.9 Kg/m^2^; Overweight: BMI: 25–29.9 Kg/m^2^; and Obese: BMI>30 Kg/m^2^)

## Results

A comparison of the IHDS-2 data and census 2011 (S1 Table) indicates that about two-third of population lived in the rural areas. While working population aged 15–64 years constituted 65%, children aged 0–14 years constituted about 28–29% of the total population in both census and IHDS survey. The composition of census and survey population was again similar with respect to marital status, religion and caste, it differed slightly with respect to female literacy rate; rate being marginally higher in census (65.5%) compared to survey (61.3%).

Table 1 presents prevalence rate (per 1000 population) for diagnosed and reported cases of asthma in India and sub-geographies. Generally speaking, prevalence rate for reported cases of asthma was 4 to 9 times higher than the rate observed for diagnosed cases for India and its sub-geographies. The prevalence rate varied from 54.9 per 1000 population for reported cases and 9.1 for diagnosed cases for India as a country. The prevalence rates were usually higher in the rural areas compared to urban areas. The difference in prevalence rates was much wider between richer and poorer states, the rates being typically higher in the poorer states. With regards to the distribution of the cases, while reported cases were found more in the poorer states, diagnosed cases were more in the richer states. The northern states of the country showed higher prevalence of reported asthma cases followed by the states in central India. In cases of diagnosed cases, southern states exhibited higher prevalence rate. The north-eastern states have shown lower prevalence rates for both reported and diagnosed cases.

**Table 1:** Prevalence rate of asthma for diagnosed cases** and reported cases*, India and sub-region, 2011–12.

Geographies	Prevalence rate (per 1000)		%age distribution (N = 204,568)		
	Reported* (N)	Diagnosed (N)	Reported*	Diagnosed	Population
**India**	54.9 (11,229)	9.1 (1,855)	-	-	-
Rural	59.8 (8,070)	9.5 (1,286)	71.9	69.3	66.0
Urban	45.4 (3,159)	8.2 (569)	28.1	30.7	34.0
Poorer States^a^	73.5 (6,073)	9.9 (816)	54.1	44.0	40.4
Richer States^b^	42.3 (5,156)	8.5 (1,039)	45.9	56.0	59.6
Northern states^c^	83.3 (4,801)	9.8 (565)	42.8	30.5	28.2
Southern states^d^	40.2 (1,694)	10.3 (432)	15.1	23.3	20.6
Eastern states^e^	54.0 (1,812)	8.3 (279)	16.1	15.0	16.4
Western states^f^	30.3 (1,244)	7.4 (304)	11.1	16.4	20.1
Central states^g^	66.4 (1,426)	11.7 (251)	12.7	13.5	10.5
North-Eastern states^h^	28.8 (252)	2.7 (24)	2.2	1.3	4.3

* reported case includes both diagnosed cases as well as cases having short breadth

**Dignosed cases includes only those cases who were ever diagnosed with asthma by doctor

***a*** Poorer states include 9 EAGA states namely: Bihar, Jharkhand, Chhattisgarh, Madhya Pradesh, Uttar Pradesh, Uttarakhand, Orissa, Rajasthan, Assam

***b*** Richer states include Non-EAGA states namely: Jammu and Kashmir, Himachal Pradesh, Punjab, Andhra Pradesh, Karnataka, Kerala, Tamil Nadu, West Bengal, Gujarat, Goa, Maharashtra, Madhya Pradesh, Sikkim, Nagaland, Meghalaya, Tripura, Mizoram, Arunachal Pradesh, Manipur

***c*** Northern states includes states namely: Jammu and Kashmir, Himachal Pradesh, Punjab, Uttar Pradesh, Uttarakhand

***d*** Southern states includes states namely: Andhra Pradesh, Karnataka, Kerala, Tamil Nadu, Pondicherry

***e*** Eastern states includes states namely: Bihar, West Bengal, Orissa, Jharkhand

***f*** Western states includes states namely: Rajasthan, Gujarat, Goa, Maharashtra, Daman and Due, Dadar and Nagar Haveli

***g*** Central states includes states namely: Madhya Pradesh, Chhattisgarh

***h*** North-Eastern states includes states namely: Assam, Sikkim, Nagaland, Meghalaya, Tripura, Mizoram, Arunachal Pradesh.

**Fig 1:**
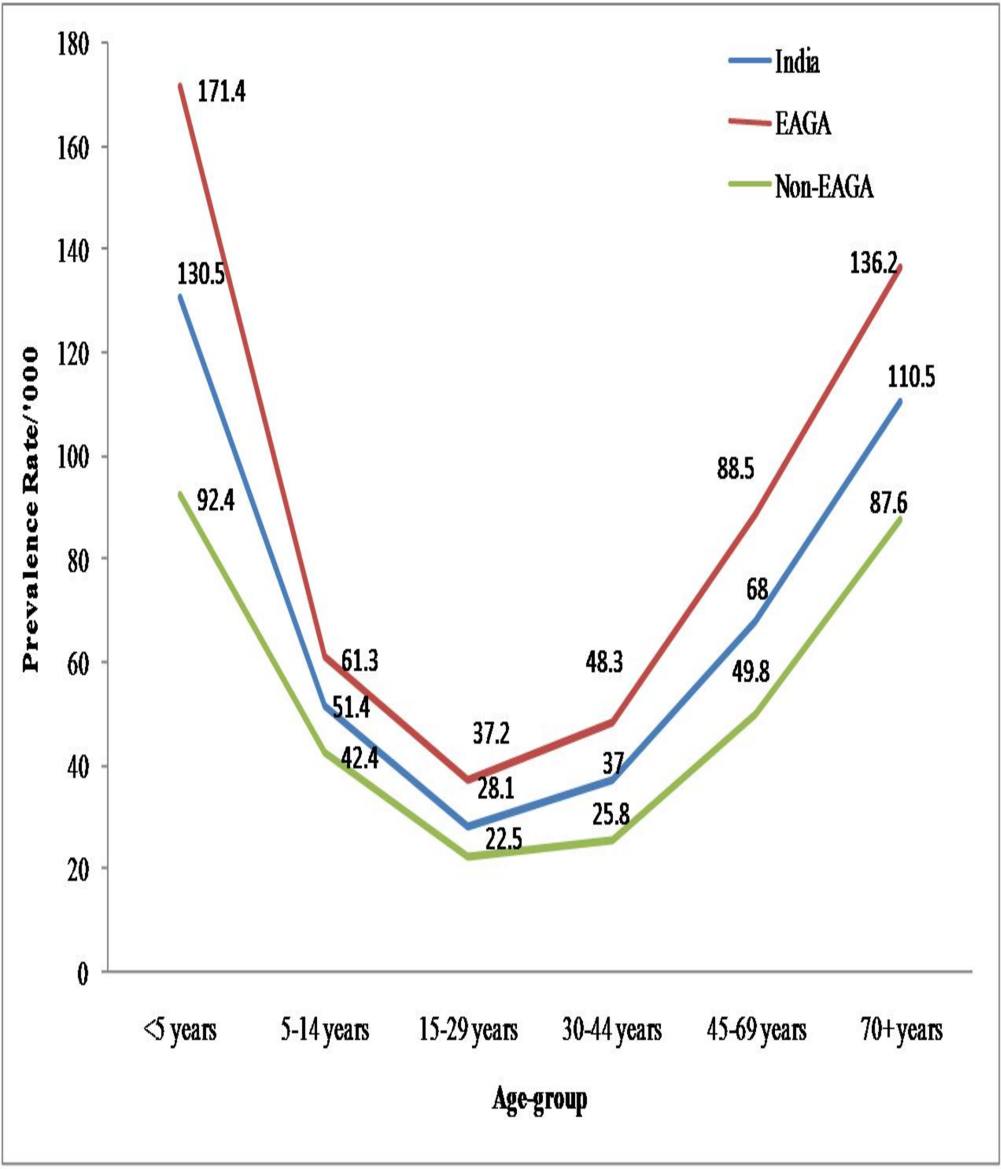
Age-specific prevalence rate of asthma for India and EAGA and Non-EAGA states.

Table 2 presents prevalence rate for reported cases of asthma by selected socio-economic-demographic and life-style related characteristics. Higher prevalence rate of asthma was observed in the rural areas compared to urban areas (59.8 versus 45.4 per 1000 population); for females than males (57.7 versus 52.0 per 1000 population). Fig 1 suggests that the age-specific asthma prevalence rates for reported cases showed U-shape curve; maxima at younger ages (below 5-years; 130.5 per 1000 population) and older ages (above 65-years; 110.5 per 1000 population). Wider difference was noted in the age-specific prevalence rate for richer and poorer states; differences narrowing at ages 15 to 29 years and again widening beyond age 29-years. For people who had zero years of schooling, the prevalence rate for asthma was thrice or more than the rate observed for those who had completed 11 or more years of schooling (87.9 versus 27.3 per 1000). Higher levels of reported cases of asthma were observed for people from lower socio-economic strata compared to those from higher socio-economic strata as measured by household wealth quintile. For example, for every 1000 people, there were about 85 people who reported asthma compared to just about 40 from richest wealth quintile. Likewise, smokers showed higher prevalence rate than the non-smokers (68.0 versus 53.8 per 1000 population). With respect to BMI, prevalence rate was significantly higher for underweight people and obese population compared to those with normal BMI (77.2 and 67.0 versus 52 per 1000 population). People living in households using unclean fuel reported 1.5 times the prevalence rate of those living in households using clean fuel only(59.4 versus 38.8 per 1000 population).The prevalence rate of reporting asthma cases was higher for Muslim population followed by Hindu population (60.2 and 53.8 per 1000 population). Scheduled tribe showed lower prevalence rate (42.3 per 1000 population), while it was higher for scheduled castes (60.2 per 1000 population).

**Table 2:** Prevalence rate and percent distribution of asthma cases by socio-economic, demographic and life-style characteristics of population, India, 2011–12.

Attributes	Prevalence rate per 1000 (N)	%age distribution of population reported	
		Asthma	No Asthma
**Sex**			
Male	52.0 (5,312)	47.3	50.0
Female	57.7 (5,917)	52.7	50.0
**Age-group**			
Less than 5 years	130.5 (2,221)	19.8	7.7
5–14 years	51.4 (2,073)	18.5	19.8
15–29 years	28.1 (1,551)	13.8	27.8
30–44 years	37.0 (1,499)	13.4	20.2
45–65 years	68.0 (2,891)	25.8	20.5
65+ years	110.5 (994)	8.9	4.1
**Marital status**			
Married	48.5 (4,797)	42.7	48.7
Never married	57.3 (5,229)	47.2	45.1
Others^#^	85.8 (1,132)	10.1	6.2
**Completed year of schooling**			
11 years and above	27.3 (849)	7.9	16.2
5–10 years	35.6 (2,235)	20.9	32.3
1–5 years	52.5 (2,281)	21.3	22.0
0 years^+^	87.9 (5,325)	49.8	29.5
**Smoke**^**++**^			
No	53.8 (10,166)	90.5	92.5
Yes	68.0 (1,063)	9.5	7.5
**Chew tobacco**^**+++**^			
No	53.9 (9,851)	87.7	89.4
Yes	63.0 (1,378)	12.3	10.6
Drink alcohol			
No	55.1 (10,617)	94.5	94.1
Yes	51.0 (612)	5.5	5.9
**Vegetarian**			
Yes	55.1 (3,089)	27.5	27.5
No	55.0 (8,133)	72.5	72.5
**Nutritional Status**			
Normal Weight	52.0 (2,934)	33.8	41.8
Underweight	77.2 (4,598)	53.0	43.0
Overweight	51.4 (771)	8.9	11.1
Obese	67.0 (373)	4.3	4.1
**Wealth quintile**			
Middle	52.5 (2,303)	20.8	20.0
Poorest	84.9 (2,441)	20.6	20.0
Poor	65.3 (2,470)	21.1	19.9
Rich	46.0 (2,080)	19.9	20.0
Richest	39.5 (1,928)	21.8	13.6
**Type of fuel use**^**$**^			
Clean only	38.8 (1,726)	15.4	22.1
Others	59.4 (9,503)	84.6	77.9
**Hours burning stove**			
Less than 3 hours	53.2 (4,002)	35.9	37.2
3 hours and more	56.1 (7,150)	64.1	62.8
**Religion**			
Hindu	53.8 (8,815)	22.0	18.3
Muslim	63.4 (1,761)	20.5	21.5
Others^&^	50.6 (653)	5.8	6.3
**Caste[13]**^**^**^			
General	53.3 (3,179)	28.3	29.3
Other Backward Class	56.1 (4,706)	42.0	41.1
Scheduled Castes	60.2 (2,599)	23.2	21.0
Scheduled Tribe	42.3 (735)	6.6	8.6
**Place of residence**			
Rural	59.8 (8,070)	71.9	65.7
Urban	45.4 (3,159)	28.1	34.3
**Total**	54.9 (11,229)	-	-

^#^Includes widow, separated and divorced

^+^0 years of schooling also includes those who never attended school

^++^includes cigarette and *biddi* both and presently smoking or ever smoked were categorised as yes and never as no

^+++^presently using and ever used categorised as yes and never as no

^$^Clean only includes LPG and Others includes firewood, crop residual, cow dung cake, coal and kerosene which were used for any purpose

^&^includes Sikh, Christian, Jain, Buddhism and others

^^^ Caste system is a sort of social class system in which people are classified based on culture and occupation.

Table 3 presents results of the multi variable logistic regression. Since model-4 present analysis combining all predictors, in this section we discuss results of this model only. However, results for other models are included in the table. In the simple bi-variate analysis, all the variables considered showed significant differentials across sub-groups of the population. As may be seen from this table, this situation does not remain true when we run full model (Model 4). Compared to children below 5-years of age, odds of reporting asthma were nearly three times lower for people aged 15–29 years (OR: 0.31;95% CI: 0.26–0.36) and 5–14 years (OR: 0.34; 95% CI: 0.30–0.39). The odds of reporting asthma increased with decline in completed years of schooling; reaching to a high level at 1.41 (95% CI: 1.21–1.64) for those who had zero years of schooling compared to those with 11 or more years of schooling. Compared to middle quintile of wealth quintile, the odds of reporting asthma was higher for poorest (OR: 1.61; 95% CI: 1.44–1.79) and poor quintile (OR: 1.21; 95% CI; 1.09–1.35) and lower for richest quintile (OR: 0.81; 95% CI: 0.72–0.92). Smokers of cigarette or *biddi* were significantly more likely to report asthma than the non-smokers. The smokers were 34% more likely to report asthma (95% CI: 1.17–1.55).Compared to people with normal weight, odds of reporting asthma was significantly higher among those with underweight (OR: 1.31; 95% CI: 1.19–1.42). People living in the household using unclean fuels exhibited higher odds ratio (OR: 1.21; 95% CI: 1.08–1.34) than those living in the household using clean fuel only. Unadjusted odds ratio and prevalence rate of reporting asthma for various unclean fuel are presented separately in supplementary table (data in S3 Table). With each additional hour of using stove (*chulha)*, the odds of reporting asthma increased by nearly 4% (OR: 1.04; 95% CI: 1.02–1.06). The odds of reporting asthma were 26% higher for Muslims compared to the Hindus. Finally, sex and place of residence did not emerge as significant predictors of reporting asthma in the full model (unlike what was observed in the bi-variate analysis).

**Table 3:** Adjusted odds ratios of reporting asthma by demographic, socio-economic, and lifestyle characteristics, India, 2011–12.

Attributes	Model 1: Demographic	Model 2: Life-Style	Mode 3: Socio-Economic	Model 4: Combine
	OR (95% CI)	OR (95% CI)	OR (95% CI)	OR (95% CI)
**Sex**				
Male	1.00			1.00
Female	1.04 (0.97–1.11)			1.04 (0.96–1.13)
**Age**				
Less than 5 years	1.00			1.00
5–14 years	0.43 (0.39–0.48)*			0.34 (0.30–0.39)*
15–29 years	0.32 (0.28–0.37)*			0.31 (0.26–0.36)*
30–44 years	0.39 (0.33–0.45)*			0.40 (0.32–0.49)*
45–69 years	0.66 (0.56–0.77)*			0.66 (0.54–0.81)*
69+ years	1.06 (0.87–1.31)			1.18 (0.89–1.55)
**Marital Status**				
Married	1.00			1.00
Never married	1.10 (0.97–1.24)			1.09 (0.92–1.29)
Others^#^	1.04 (0.92–1.18)			1.15 (0.98–1.34)**
**Completed year of Schooling**				
11 years and above	1.00			1.00
6–10 Years	1.29 (1.15–1.44)*			1.12 (0.97–1.28)
1–5 Years	1.69 (1.51–1.91)*			1.24 (1.07–1.44)*
0 Years^+^	2.17 (1.94–2.43)*			1.41 (1.21–1.64)*
**Smoke**^**++**^				
No		1.00		1.00
Yes		1.67 (1.44–1.93)*		1.34 (1.17–1.55)*
**Chew Tobacco**^**+++**^				
No		1.00		1.00
Yes		1.30 (1.17–1.44)*		1.07 (0.95–1.19)
**Drink alcohol**^**+++**^				
No		1.00		1.00
Yes		0.61 (0.52–0.73)*		0.73 (0.62–0.86)*
**Vegetarian**				
Yes		1.00		1.00
No		0.93 (0.86–1.01)***		0.85 (0.78–0.93)*
**Nutritional status**				
Normal Weight		1.00		1.00
Underweight		1.64 (1.53–1.76)*		1.31 (1.19–1.42)*
Overweight		1.04 (0.92–1.18)		1.10 (0.97–1.25)
Obese		1.32 (1.14–1.53)*		1.14 (0.98–1.34)***
**Wealth Quintile**				
Middle			1.00	1.00
Poorest			1.89 (1.72–2.07)*	1.61 (1.44–1.79)*
Poor			1.25 (1.15–1.37)*	1.21 (1.09–1.35)*
Rich			0.86 (0.79–0.94)*	0.94 (0.84–1.05)
Richest			0.75 (0.68–0.83)*	0.81 (0.72–0.92)*
**Religion**				
Hindu			1.00	1.00
Muslim			1.19 (1.10–1.28)*	1.26 (1.14–1.39)*
Others^&^			1.24 (1.06–1.44)*	1.36 (1.14–1.64)*
**Caste[13]**^**^**^				
General			1.00	1.00
Other Backward Class			1.02 (0.95–1.09)	0.96 (0.88–1.05)
Scheduled Castes			0.99 (0.91–1.08)	0.98 (0.88–1.09)
Scheduled Tribe			0.59 (0.52–0.67)*	0.63 (0.54–0.73)*
**Type of fuel used**^**$**^				
Clean only			1.00	1.00
Others			1.26 (1.15–1.37)*	1.21 (1.08–1.34)*
**Hours burning stove**			1.03 (1.01–1.04)*	1.04 (1.02–1.06)*
**Place of residence**				
Rural			1.00	1.00
Urban			1.01 (0.95–1.08)	0.98 (0.91–1.06)

*significant at p<0.01

**significant at p<0.05

***not significant at p<0.05 but significant at p<0.1

^#^Includes widow, separated and divorced

^+^0 years of schooling also includes those who never attended school

^++^includes cigarette and *biddi* both and presently smoking or ever smoked were categorised as yes and never as no

^+++^presently using and ever used categorised as yes and never as no

^&^includes Sikh, Christian, Jain, Buddhism and others

^^^Caste system is a sort of social class system in which people are classified based on culture and occupation

^$^Clean only includes LPG and Others includes firewood, crop residual, cow dung cake, coal and kerosene which were used for any purpose

Table 4 presents total number of estimated cases of asthma, Population Attributable Fraction (PAF) and avoidable number of cases of asthma for India. In the year 2015, an estimated of nearly 65 million people in India suffered from asthma. Of these 65 million asthma cases, the burden was one of the highest at 80% (~53.1M) for those living in the households using firewood as fuel followed by 78% (~51.6M) among those living in households using kerosene and 52% (~34.2M) for those living in households using cow-dung-cakes. The analysis of PAFs indicates that almost 24.7% of the asthma cases are attributable to use of firewood alone followed by dung cake and kerosene (~17% each).This translates into 16 million avoidable asthma cases resulting due to firewood use and 11 million avoidable cases each as a result of kerosene and dung cake use. With respect to completed years of schooling, analysis suggests that compared to individuals who had completed 11 or more years of schooling, the burden is higher for those with no schooling (50.7%; 32.5M) leading to 21% cases attributable due to less schooling. This translates into nearly 13.5M avoidable cases if everyone completes 11 or more years of schooling. The burden of asthma was around six times higher among underweight people (40.7M; accounting for 62% of the total burden), compared to those with normal weight (6.8M). Among the underweight people, around 17.3% of the cases are attributable to underweight compared to those with normal weight, translating into around 11 million avoidable cases. The burden of asthma among individuals who smoke cigarette/*biddi* was 5.5 million which constitute nearly 8% of the total burden. The analysis of PAFs indicate that almost 2% of the asthma cases were attributable to smoking cigarette/*biddi*, leading to nearly 1.3 million avoidable cases that could be avoided by eliminating cigarette/*biddi* consumption. The burden of asthma was 33M for people living in households using hand pump as a source of water. About 17% of the asthma cases are attributable to use of hand pump; this translates into 11 million avoidable cases of asthma.

**Table 4:** Estimated number of asthma cases, Population attributable fraction (PAF), and avoidable cases as a result of improvement in modifiable risk factors, India, 2011–12.

Attributes	Number of asthma cases	PAF	Estimated avoidable cases
**Completed years of schooling (R = 11 years and more)**			
0 Years^+^	32,580,000	21.1	13,586,000
1–5 Years	13,954,000	10.54	6,783,000
6–10 Years	13,660,000	7.39	4,756,000
**Smokers (R = No)**^**++**^			
Smokers	5,573,000	1.98	1,275,000
**Tobacco chewers (R = No)**^**+++**^			
Tobacco chewers	8,155,000	1.77	1,139,000
**Nutritional status (R = Normal weight)**			
Underweight	40,775,000	17.30	11,137,000
Overweight	6,834,000	0.10	64,000
Obese	3,310,000	0.96	618,000
**Type of house (R = *Pakka* house)**^**@**^			
Semi-*pakka* house	42,601,000	6.33	4,075,000
*Kachha* house	2,901,000	0.91	586,000
**Electricity in house (R = Yes)**			
No electricity	12,875,000	8.05	5,182,000
**Type of fuel used (R = Not using)**^**&**^			
Firewood	53,083,000	24.72	15,914,000
Dung cake	34,169,000	16.89	10,873,000
Crop residual as	18,976,000	6.74	4,339,000
Kerosene	51,564,000	16.54	10,648,000
**Water source (R = Piped water)**			
Tube well	6,112,000	1.02	657,000
Hand pump	33,000,000	17.15	11,041,000
Open well	4,780,000	1.04	670,000

^+^0 years of schooling also includes those who never attended school

^++^includes cigarette and *biddi* both and presently smoking or ever smoked were categorised as yes and never as no

^+++^presently using and ever used categorised as yes and never as no

^&^used for any purpose and reference category is not using particular fuel

^@^is computed using three variables: type of floor, type of roof and type of walls.

## Discussion

The present study uses IHDS-2, a nationwide cross-sectional survey data and observed an increasing trend in the prevalence rate of reporting asthma between 2005 and 2012. This is noted for country as a whole and also its sub-geographies–urban versus rural residence, richer states versus poorer states; six regions of the country. The prevalence rate for reporting asthma was nearly 4 to 9 times higher than the prevalence rate for ever diagnosed cases of asthma and the prevalence rate has increased from 41.9 per 1000 population in year 2004–05 to 54.9 per 1000 population in the year 2011–12. It is important to note that such differences may be the result of under-reporting due to lack/poor knowledge about the disease in the community [14–16].

Most of the studies on asthma in India, like Aggarwal et al. (2006), define asthma as cases ever diagnosed with asthma or received treatment for asthma [8]. In the present study we have also included additional symptoms cough with short breath for which information is available in the survey data. Thus the definition adopted in the present study for may have led to higher prevalence rates than the rates observed in previous studies based on general population [8, 15] which ranged between 20 to 35 per 1000 population. Nonetheless, Brashier et al. (2012) have found prevalence rate for self-reporting of asthma as 100 per 1000 population for those residing in slums of Pune city, India [16]. In another study conducted in Mumbai, the prevalence rate of asthma in adult was estimated at 35 and 170 per 1000 population for doctor diagnosed cases and using broad definition [17] which includes all such cases as asthma attack, bronchial reactivity, allergic symptoms, and specific allergy.

Around 65M cases and 82,000 deaths of asthma were estimated for the year 2015 in India (data not shown). Asthma deaths accounted for nearly one percent of the total deaths in India in 2015. The estimated total burden of asthma was nearly two and half times higher than burden estimated by Agrawal, Pearce, and Ebrahim in (2013) using NFHS-3 data [6]. This difference in the burden can be attributed to lower prevalence rate of reporting asthma in their study which was around two and half times lower than our estimates. Such differences may be explained by the fact that the data sets used in two studies are not only different but also refer to different time points, the NFHS-3 and IHDS data are almost six years apart. It needs to be mentioned that our estimates using earlier round of the IHDS (which was around the same time as NFHS-3) were also on the higher side simply because of difference in the definition used for identifying asthma cases (S2 Table).

The morbidity burden due to asthma was higher for people who have completed fewer years of schooling, who smoke, those who were under-weigh and those residing in households where unclean fuel was used and/or where hand pump is used as water source. Solid fuel users—firewood, crop residual and cow dung cake, were 21% more likely to report asthma. This finding is consistent with that of existing literature where a significant association between exposure to unclean fuel and asthma reporting has been documented [15]. Patra et al. (2016) have also found linear relationship between smoking behaviour and prevalence of asthma. However, in a study among slum dwellers of Pune city, India, Brashier et al. (2012) did not find any association between exposure to unclean fuel and asthma [6]. Although there is a decrease in household air pollution from solid fuels in Southeast Asia, majority of the households in India continue to use solid fuels for household use which leads to indoor air pollution, which accounts for approximately 3.5 million deaths and 4.5% of the global daily-adjusted-life-years (DALY) in 2010 [18]. The on-going schemes like *Pradhan MantriUjjwala Yojana* (PMUY) in India which aim at safeguarding health of the women and children by providing households with a clean cooking fuel (that is, LPG) can be useful in preventing respiratory diseases and thereby asthma [19]. The monitoring and assessment of such schemes in terms of reduction of indoor pollution or its impact on respiratory diseases due to indoor pollution however is a must.

Regional differences in the prevalence rate of asthma were observed in the present study; higher in the rural areas than in urban areas, a finding that is consistent with the past cross-sectional surveys based study [6]. Contrary to this, Aggarwal et al. (2006) in a multi-centric study undertaken in Chandigarh, Delhi, Bangalore and Kanpur, found higher prevalence rate of asthma in the urban area which was similar to studies from the developed countries [20]. According to International Study of Asthma and Allergies in Childhood (ISSAC) study in Britain, the prevalence of asthma was higher in non-metropolitan area than the metropolitan areas [21]. The higher asthma prevalence rate in our study can be attributed to greater use of solid fuels in rural area. The study noted significant differences in the prevalence rate of reporting asthma in the sub-geographies of India. Significant differences in the prevalence rate of asthma were observed between poorer and richer states with lower in the latter. The six geographic regions showed wide variation in the asthma prevalence rates; higher rates in northern India and lower in the north-eastern states. This finding was contrasting to what was observed by Agrawal, Pearce, and Ebrahim (2013) [6], where they found higher rates in the eastern India and lowest in the central India. These results are similar to the results for IHDS-1 where higher rates were observed in the eastern states and lower in central (S2 Table). The study also notes that the percentage distribution of reported cases were higher than diagnosed cases in northern, eastern and north-eastern states, whereas, share of diagnosed cases were higher in southern, western and central states. Nine poorer states of EAGA group have higher share of reported cases but lower share of diagnosed cases compared to the richer states. This may be due to lack of awareness and knowledge about asthma. In a study conducted in Delhi, it was found that around 40% of the patients were not informed about the disease and only 10% had undergone lung function tests previously [22].

The study further notes risk factors associated with prevalence of reporting asthma–such as age, years of schooling, socio-economic status, smoking behaviour, and body mass index etc. The study notes that the prevalence rate for asthma varies by socio-economic and demographic attributes of the individuals; with higher rates observed for people living in rural areas, poorer states and children and older people compared to their respective counterparts. Further, the rates are higher for people from lower economic strata, fewer years of schooling, scheduled castes and Muslims compared to their respective counterparts. Further analysis for variables used in computation of wealth quintile (S3 Table) and also separate analysis stratified by rural-urban and male-female was performed to examine differential in the prevalence rate of reporting asthma (S4 and S5 Tables) by various socio-economic characteristics.

Age of the person emerged as another prominent factor for asthma reporting; with higher rates among children below age 5-years and 70 years and older, forming a U-shape curve. There was no notable difference in the shape of the age specific prevalence rates for both EAGA and Non-EAGA states; however, the levels were significantly higher for EAGA states for each age group. Haby et al. (2001), Hoskins et al. (2000) and Holland (1975) too noted higher prevalence rate among children in low income as well as high income countries [23–25]. In a systematic review of 15 epidemiological studies on the development of asthma in India, it was observed that asthma prevalence among children aged 13–14 years was lower than the rate for children aged 6–7 years [26]. Around one-third of the children reports first attack of asthma before the age of 2 to 5 years [25]. There is a lack of consensus as far as sex as a risk factor is concerned; Some literatures suggests male at higher risk [25,26,27] while other suggests females at higher risk [6]. However, in the present study the prevalence rate was higher for females; however, this disadvantage disappears in the logistic regression analysis. This finding was similar to the finding by Kumar et al. (2014) [28]. However, male-female differential was noted when stratified by rural-urban, with significantly higher risk of reporting asthma for females in urban areas while difference was insignificant in rural areas. It was further found that the risk of asthma was higher among poor people as the likelihood of reporting asthma decreased with an improvement in the wealth quintile. This finding is consistent with what is available in the literature [27,29].

Nutritional status emerged as one of the key modifiable risk factor with significantly higher burden of asthma. The prevalence rate of asthma by body weight shows U-shape curve indicating higher rates among underweight and obese but lower among normal weight and overweight. Similar results were found by Agrawal, Pearce, and Ebrahim in 2013 using NFHS-3 data [6]. Using World Health Survey, Patra et al. (2016) found the quadratic relationship between asthma and BMI with higher prevalence for underweight and obese people [30]. The percent avoidable cases due to overweight were around 19 percent and for underweight it was 27 percent. Seidman et al. (1991) found that low birth weight was significantly associated with prevalence of asthma among adolescents and the group with low birth weights had increased risk of developing asthma by 17 years of age [31]. Contrary to this, Von Kries et al. (2001) found that doctors diagnosed prevalence rate of asthma was higher among obese and overweight children and more confined to girls than boys [32]. Also E. Huovinen, J. Kaprio, and M. Koskenvu (2003), in a cohort study of 10,597 Finnish adults, have found that obesity increases the risk of asthma [33].

In the study, it was also observed that people from Muslim community were more likely to report asthma than people from Hindu community. Similar result was observed by Agrawal, Pearce, and Ebrahim (2013) and Subramanian et al. 2007 [6,34]. This difference in the prevalence might be attributed to dietary habits. The association of asthma and dietary pattern is established in literature. Aggarwal, Pearce, and Ebrahim (2013) have found that the risk of asthma is higher among non-vegetarian people compared to those who are only vegetarian [6]. In the present study, it was found that Muslim community have higher proportion of non-vegetarian people. And association between reporting of asthma and non-vegetarian diet was found in bivariate analysis (data not shown). Also, in the study it was found that negative association exists between ST caste and asthma as compared to general caste. The association is not well discussed in literature even similar lower prevalence was found by Subramanian et al. 200 [34]. Further community specific research is needed to explore the reason for lower likelihood of reporting asthma in ST caste and also to examine the effect of dietary habit on prevalence of asthma in India.

The present study although analyses most recent data available on the topic, however, there are a few limitation as the analysis draws inferences from the data that is cross-sectional in nature and also lack information on all the necessary aspects/dimension/processes related to asthma in India. As the information was collected from the household head for all the family members, there is a possibility for recall and response biases. Further, the cases of asthma are primarily identified on symptoms. The asthma cases have been defined based on two-types of information–persons ever diagnosed based on a single question and only one symptom of cough with short breath. The second aspect become even more important as asthma is not a well-defined disease thus call for inclusion of more than one symptom is required.

## Conclusion

The present analysis notes a rising pattern in the prevalence rates of Asthma at national as well as sub-national levels. This means, that with this trend, the burden of asthma in India is only going to higher and higher in the years to come. This may be even more alarming since we are still in dynamic phase of population growth, hence are expected to grow further before reaching stable level. The statistical analysis carried out indicates a very strong positive association between asthma and solid fuel use and source of light for the households. The households using solid fuel for purposes such as cooking, heating and lighting (in the absence of electricity) had higher risk of reporting an asthma case. This calls for a greater emphasis and wider reach of the on-going program of providing clean fuel (LPG) for cooking under the PMUY to the under-privileged poor households in both urban and rural areas of the country. This would ensure healthier indoor environ and hence may reduce prevalence of asthma and thereby its burden to the nation. The analysis also confirms higher prevalence of asthma among non-literate or poorly literate members. This becomes an important finding as the awareness of harms of using solid fuel was extremely poor among the surveyed households. Thus, an initiation of a program that exclusively aims at creating awareness in the masses on indoor air-pollution and its impact on respiratory health in general and that of asthma in particular may be a way forward to combat the rising asthma prevalence.

As pointed out previously, India lacks data that comprehensively investigates on various issues associated with asthma in the country. In view of rising trends in asthma prevalence as well its regional pattern, it is very important for India to make sincere efforts in generating database evidence on asthma. It will help plan more effectively if surveys on a regular interval were undertaken in India on asthma. Further, the national level data has less value as the prevalence and patterns of asthma vary significantly at sub-national level. Not only this, the sub-national pattern has also indicated change over time. So a longitudinal dataset may be more useful to investigate on such insights.

## Supporting information

S1 Table Percentage distribution of population for IHDS-2 and census 2011, India. (PDF)

S2 Table Prevalence rate of asthma for diagnosed cases** and reported cases*, India and sub-region, 2004–05. (PDF)

S3 Table Prevalence rate and unadjusted odds ratio of asthma for selected socio-economic, demographic and environmental characteristics of population, India, 2011–12. (PDF)

S4 Table Rural-urban differential in prevalence rate and unadjusted odds ratio of asthma by socio-economic, demographic and environmental characteristics India, 2011–12. (PDF)

S5 Table Gender differential in prevalence rate and unadjusted odds ratio of asthma by socio-economic, demographic and environmental characteristics, India, 2011–12. (PDF)
