# Supplementary material for: Patterns, factors associated and morbidity burden of asthma in India
Source: PLoS One. 2017 Oct 26;12(10):e0185938. doi: 10.1371/journal.pone.0185938 (PMC5657621; doi:10.1371/journal.pone.0185938)
Supplement: S1 Table — (PDF) [file pone.0185938.s001.pdf]

Attributes	Percentage distribution of population	
	IHDS-2 (2011-12)	Census 2011
**Place of residence**		
Rural	66.0	69.0
Urban	34.0	31.0
**Sex**		
Male	50.0	51.5
Female	50.0	48.5
**Age**		
Less than 5 years	8.3	7.4
6-14 years	19.7	21.5
15-29 years	18.9	27.6
30-44 years	27.9	20.4
45-65 years	18.0	16.7
65+ years	7.2	4.4
**Literacy rate**	68.5	74.0
**Marital Status**		
Never Married	45.2	47.1
Married	48.3	47.9
Others	6.5	5.0
**Religion**		
Hindu	80.1	79.8
Muslim	13.6	14.2
Others	6.3	6.0
