# Supplementary material for: Patterns, factors associated and morbidity burden of asthma in India
Source: PLoS One. 2017 Oct 26;12(10):e0185938. doi: 10.1371/journal.pone.0185938 (PMC5657621; doi:10.1371/journal.pone.0185938)
Supplement: S2 Table — (PDF) [file pone.0185938.s002.pdf]

Regions	Prevalence rate (per 1000)		%age distribution (N=215,754)		
	**Reported (N)**	**Diagnosed (N)**	**Reported**	**Diagnosed**	**Population**
**India**	41.9 (9,049)	5.5 (1,197)	-	-	-
Rural	45.2 (6,484)	5.9 (853)	71.6	71.3	66.4
Urban	35.4 (2,565)	4.8 (344)	28.4	28.7	33.6
Poorer States[a]	53.3 (4,463)	5.7 (480)	49.3	40.1	39.3
Richer States[b]	35.0 (4,586)	5.5 (717)	50.7	59.9	60.7
Northern states[c]	53.3 (3,134)	4.6 (271)	34.6	22.6	27.3
Southern states[d]	23.7 (1,126)	6.7 (319)	12.4	26.7	22.0
Eastern states[e]	64.7 (2,288)	7.3 (257)	25.3	21.5	16.4
Western states[f]	29.3 (1,249)	5.9 (253)	13.8	21.1	19.8
Central states[g]	45.4 (1,000)	4.3 (94)	11.1	7.9	10.2
North-Eastern states[h]	26.7 (252)	1.0 (03)	2.8	0.3	4.4

* reported case includes both diagnosed cases as well as cases having short breadth;

**Dignosed cases includes only those cases who were ever diagnosed with asthma by doctor;

a Poorer states include 9 EAGA states namely: Bihar, Jharkhand, Chhattisgarh, Madhya Pradesh, Uttar Pradesh, Uttarakhand, Orissa, Rajasthan, Assam;

b Richer states include Non-EAGA states namely: Jammu and Kashmir, Himachal Pradesh, Punjab, Andhra Pradesh, Karnataka, Kerala, Tamil Nadu, West Bengal, Gujarat, Goa, Maharashtra, Madhya Pradesh, Sikkim, Nagaland, Meghalaya, Tripura, Mizoram, Arunachal Pradesh, Manipur;

c Northern states includes states namely: Jammu and Kashmir, Himachal Pradesh, Punjab, Uttar Pradesh, Uttarakhand;

d Southern states includes states namely: Andhra Pradesh, Karnataka, Kerala, Tamil Nadu, Pondicherry;

e Eastern states includes states namely: Bihar, West Bengal, Orissa, Jharkhand;

f Western states includes states namely: Rajasthan, Gujarat, Goa, Maharashtra, Daman and Due, Dadar and Nagar Haveli;

g Central states includes states namely: Madhya Pradesh, Chhattisgarh;

h North-Eastern states includes states namely: Assam, Sikkim, Nagaland, Meghalaya, Tripura, Mizoram, Arunachal Pradesh.
