# Supplementary material for: Patterns, factors associated and morbidity burden of asthma in India
Source: PLoS One. 2017 Oct 26;12(10):e0185938. doi: 10.1371/journal.pone.0185938 (PMC5657621; doi:10.1371/journal.pone.0185938)
Supplement: S3 Table — (PDF) [file pone.0185938.s003.pdf]

Covariate	Prevalence per (1000)	Crude OR (95% CI)
**Type of house[@]**		
Kachha House	74.4 (323)	1.00
Semi-*Pakka* House	56.7 (6,944)	0.68 (0.58-0.80)*
Pakka House	50.9 (3,962)	0.58 (0.49-0.69)*
**Electricity in house**		
No	86.8 (2,167)	1.00
Yes	50.6 (9,057)	0.52 (052-0.56)*
**Motor vehicle**		
No	61.4 (8,386)	1.00
Yes	41.9 (2,837)	0.62 (0.59-0.66)*
**Cooking place**		
In living area	67.4 (3,104)	1.00
Outdoors	64.3 (2,416)	1.50 (1.40-1.61)*
Separate Kitchen	47.4 (5,702)	1.46 (1.36-1.57)*
**Stove type**		
Others/Not Biomass	41.9 (3,003)	1.00
Open fire	65.3 (2,173)	1.83 (1.67-2.01)*
Traditional stove	64.3 (5,384)	1.73 (1.64-1.84)*
Improved stove	43.6 (640)	1.35 (1.19-1.53)*
**Firewood as fuel**		
No	413 (2,255)	1.00
Yes	69.8 (8,974)	1.78 (1.68-1.90)*
**Cow dung cake as fuel**		
No	45.6 (5,454)	1.00
Yes	67.9 (5,775)	1.89 (1.77-2.02)*
**Crop residual as fuel**		
No	51.2 (8,021)	1.00
Yes	67.9 (3,208)	1.94 (1.83-2.06)*
**Kerosene as fuel**		
No	45.8 (2,514)	1.00
Yes	58.2 (8,715)	1.69 (1.59-1.82)*
**Coal as fuel**		
No	55.2 (10,864)	1.00
YEs	46.1 (365)	1.15 (0.98-1.36)**
**LPG as fuel**		
No	63.6 (6,911)	1.00
Yes	45.0 (4,318)	0.62 (0.59-0.66)*
**Source of water**		
Piped water	44.1 (4,209)	1.00
Tube well	49.1 (1,126)	1.40 (1.23-1.59)*
Hand pump	77.8 (4,446)	1.93 (1.28-2.05)*
Open well	51.0 (860)	1.13 (1.03-1.25)*
Others	48.7 (588)	1.13 (1.01-1.28)*

[*]significant at $p<0.05$; [**]not significant at $p<0.05$ but significant at $p<0.01$; [@]is computed using three variables: type of floor, type of roof, and type of wall
