# Supplementary material for: Patterns, factors associated and morbidity burden of asthma in India
Source: PLoS One. 2017 Oct 26;12(10):e0185938. doi: 10.1371/journal.pone.0185938 (PMC5657621; doi:10.1371/journal.pone.0185938)
Supplement: S4 Table — (PDF) [file pone.0185938.s004.pdf]

Covariates	Rural		Urban	
	Prevalence per 1000 (N)	Adjusted OR (95% CI)	Prevalence per 1000 (N)	Adjusted OR (95% CI)
**Sex**				
Male	57.1 (3,834)	1.00	42.3 (1,478)	1.00
Female	62.4 (4,236)	1.01 (0.91-1.12)	48.6 (1,681)	1.17 (1.05-1.30)*
**Age-group**				
Less than 5 years	135.7 (1,653)	1.00	117.3 (568)	1.00
5-14 years	52.5 (1,476)	0.31 (0.27-0.36)*	48.9 (597)	0.45 (0.37-0.55)*
15-29 years	29.2 (1,045)	0.28 (0.23-0.35)*	26.0 (506)	0.38 (0.31-0.49)*
30-44 years	42.0 (1,076)	0.39 (0.30-0.51)*	28.4 (423)	0.43 (0.32-0.59)*
45-69 years	75.8 (2,063)	0.64 (0.49-0.83)*	54.2 (828)	0.71 (0.53-0.96)*
69+ years	122.2 (757)	1.17 (0.83-1.64)	84.7 (237)	1.20 (0.84-1.72)
**Marital status**				
Married	53.6 (3,499)	1.00	38.7 (1,298)	1.00
Unmarried	61.4 (3,750)	1.04 (0.84-1.30)	49.4 (1,549)	1.12 (0.90-1.41)
Others[#]	95.1 (820)	1.19 (0.99-1.43)***	68.2 (312)	1.02 (0.83-1.26)
**Year of schooling**				
11 years and above	27.4 (391)	1.00	27.1 (458)	1.00
6-10 years	36.9 (1,445)	1.05 (0.86-1.29)	33.6 (219)	1.16 (0.99-1.37)***
1-5 years	54.6 (1,658)	1.13 (0.91-1.39)	47.8 (623)	1.40 (1.17-1.68)*
0 years[+]	90.4 (4,250)	1.23 (1.01-1.51)**	79.4 (1,075)	1.85 (1.50-2.23)*
**Smoke**[++]				
No	58.6 (7,226)	1.00	44.9 (2,940)	1.00
Yes	72.5 (844)	1.29 (1.11-1.52)*	54.9 (219)	1.45 (1.13-1.87)*
**Chew tobacco**[+++]				
No	58.4 (6,938)	1.00	45.6 (2,913)	1.00
Yes	69.7 (1,132)	1.07 (0.94-1.23)	43.7 (246)	0.92 (0.74-1.12)
**Drink Alcohol**[+++]				
No	60.1 (7,600)	1.00	45.6 (3,017)	1.00
Yes	54.5 (470)	0.73 (0.60-0.87)*	42.0 (142)	0.77 (0.55-1.05)
**Vegetarian**				
Yes	59.8 (2,390)	1.00	43.5 (699)	1.00
No	60.0 (5,675)	0.83 (0.75-0.92)*	46.2 (2,458)	0.94 (0.83-1.06)
**Body Mass Index**				
Normal Weight	56.4 (2,141)	1.00	42.9 (793)	1.00
Underweight	80.5 (3,554)	1.32 (1.19-1.46)*	67.7 (1,044)	1.25 (1.09-1.43)*
Overweight	60.6 (454)	1.19 (1.01-1.43)**	42.3 (317)	0.98 (0.85-1.14)
Obese	76.7 (201)	1.14 (0.89-1.43)	58.3 (172)	1.17 (0.96-1.42)
**Wealth Quintile**				
Middle	51.8 (1,762)	1.00	54.9 (541)	1.00
Poorest	86.0 (2,369)	1.67 (1.48-1.88)*	59.8 (72)	0.94 (0.68-1.29)
Poor	65.5 (2,198)	1.24 (1.10-1.40)*	64.3 (272)	1.15 (0.96-1.40)
Rich	46.7 (1,138)	0.94 (0.82-1.09)	45.2 (942)	0.94 (0.82-1.09)
Richest	38.7 (598)	0.75 (0.62-0.91)*	39.9 (1,330)	0.88 (0.75-1.04)
**Type of fuel use**[$]				
Clean only	33.4 (204)	1.00	39.6 (21,522)	1.00
Others	61.0 (5,264)	1.23 (0.96-1.57)	52.6 (1,637)	1.25 (1.11-1.41)*
**Hours burning stove**				

		--		--
Less than 3 hours	57.7 (2,756)	--	45.3 (2,182)	--
3 hours and more	61.1 (5,264)		45.8 (950)	
**Hours burning stove**	--	1.05 (1.02-1.08)*	--	1.01 (0.96-1.03)
**Religion**				
Hindu	58.7 (6,539)	1.00	43.5 (2,276)	1.00
Muslim	71.8 (1,075)	1.31 (1.15-1.48)*	53.6 (686)	1.14 (1.01-1.31)**
Others&	52.8 (456)	1.38 (1.08-1.76)*	46.2 (197)	1.32 (1.09-1.61)*
**Caste^**				
General	59.0 (2,046)	1.00	45.4 (1,133)	1.00
Other Backward Class	61.1 (3,357)	0.98 (0.88-1.11)	46.5 (1,349)	0.89 (0.80-0.99)**
Scheduled Castes	65.5 (1,989)	1.01 (0.87-1.19)	47.7 (610)	0.94 (0.81-1.08)
Scheduled Tribes	45.1 (673)	0.65 (0.55-0.78)*	25.1 (62)	0.51 (0.36-0.71)*
**Total**	59.8 (8,070)	--	45.4 (3,159)	--

***significant at p<0.05; **not significant at p<0.05 but significant at p<0.1; #Includes widow, separated and divorced; +0 years of schooling also includes those who never attended school; ++includes cigarette and *biddi* both and presently smoking or ever smoked were categorised as yes and never as no; +++presently using and ever used categorised as yes and never as no;; &includes Sikh, Christian, Jain, Buddhism and others; ^Caste system is a sort of social class system in which people are classified based on culture and occupation [13], $Clean only includes LPG and Others includes firewood, crop residual, cow dung cake, coal and kerosene which were used for any purpose
