# Supplementary material for: Patterns, factors associated and morbidity burden of asthma in India
Source: PLoS One. 2017 Oct 26;12(10):e0185938. doi: 10.1371/journal.pone.0185938 (PMC5657621; doi:10.1371/journal.pone.0185938)
Supplement: S5 Table — (PDF) [file pone.0185938.s005.pdf]

	Male		Female	
**Covariates**	**Prevalence per 1000 (N)**	**Adjusted OR (95% CI)**	**Prevalence per 1000 (N)**	**Adjusted OR (95% CI)**
**Age-group**				
Less than 5 years	138.1 (1,251)	1.00	122.3 (1,006)	1.00
5-14 years	54.9 (1,158)	0.35 (0.29-0.43)*	47.6 (915)	0.32 (0.27-0.38)*
15-29 years	24.2 (660)	0.28 (0.23-0.37)*	31.8 (891)	0.31 (0.24-0.39)*
30-44 years	24.4 (480)	0.38 (0.26-0.56)*	49.0 (1,019)	0.43 (0.33-0.56)*
45-69 years	60.7 (1,272)	0.78 (0.56-1.07)	75.2 (1,619)	0.66 (0.50-0.86)*
79+ years	123.7 (527)	1.74 (1.20-2.52)*	98.6 (467)	0.86 (0.54-1.37)
**Marital status**				
Married	44.2 (2,124)	1.00	52.5 (2,673)	1.00
Unmarried	57.1 (2,939)	1.42 (1.09-1.84)*	57.5 (2,360)	1.06 (0.83-1.34)
Others[#]	94.9 (248)	1.38 (0.98-1.92)***	83.5 (884)	1.12 (0.93-1.34)
**Year of schooling**				
11 years and above	25.1 (461)	1.00	30.4 (388)	1.00
6-10 years	32.8 (1,145)	0.98 (0.80-1.21)	39.2 (1,090)	1.68 (0.96-1.41)
1-5 years	54.2 (1,210)	1.18 (0.94-1.48)	50.8 (1,071)	1.82 (0.97-1.44)***
0 years[+]	95.5 (2,188)	1.41 (1.09-1.80)*	83.3 (3,137)	1.26 (1.03-1.53)**
**Smoke[++]**				
No	50.1 (4,372)	1.00	57.0 (5,794)	1.00
Yes	63.8 (940)	1.35 (1.14-1.60)*	135.8 (123)	1.85 (1.34-2.57)*
**Chew tobacco[+++]**				
No	51.4 (4,367)	1.00	56.1 (5,484)	1.00
Yes	55.3 (945)	1.12 (0.95-1.32)	90.5 (433)	1.15 (0.96-1.37)
**Drink Alcohol[+++]**				
No	52.3 (4,735)	1.00	57.7 (5,882)	1.00
Yes	50.1 (577)	0.81 (0.67-0.97)**	72.5 (35)	0.74 (0.43-1.26)
**Vegetarian**				
No	51.3 (1,440)	1.00	58.9 (1,649)	1.00
Yes	52.5 (3,869)	0.88 (0.77-1.01)***	57.5 (4,264)	0.82 (0.73-0.92)*
**Body Mass Index**				
Normal Weight	48.7 (1,129)	1.00	54.3 (1,805)	1.00
Underweight	84.2 (2,409)	1.50 (1.31-1.72)*	70.6 (2,189)	1.16 (1.04-1.30)*
Overweight	38.1 (212)	1.17 (0.90-1.52)	59.3 (559)	1.06 (0.92-1.21)
Obese	60.3 (109)	0.95 (0.72-1.26)	70.2 (264)	1.21 (0.99-1.46)***
**Wealth quintile**				
Middle	49.6 (1,090)	1.00	55.5 (1,213)	1.00
Poorer	85.0 (1,195)	1.76 (1.49-2.07)*	84.8 (1,246)	1.49 (1.29-1.72)*
Poor	63.3 (1,187)	1.30 (1.11-1.53)*	67.3 (1,283)	1.17 (1.02-1.34)**
Rich	42.1 (950)	0.94 (0.80-1.13)	49.9 (1,130)	0.94 (0.82-1.08)
Richer	36.0 (887)	0.83 (0.68-1.02)***	43.0 (1,041)	0.78 (0.67-0.92)*
**Type of fuel use[$]**				
Clean only	35.7 (800)	1.00	41.8 (926)	1.00
Others	56.6 (4,512)	1.14 (0.95-1.37)	62.1 (4,991)	1.25 (1.10-1.43)*
**Hours burning stove**				
		--		--
Less than 3 hours	50.7 (3,405)	--	56.5 (3,813)	--
3 hours and more	55.1 (1,867)		60.7 (2,067)	
**Hours burning stove**	--	1.06 (1.03-1.11)*	--	1.02 (0.98-1.05)
**Religion**				
Hindu	51.1 (4,179)	1.00	56.4 (4,636)	1.00
Muslim	61.5 (850)	1.25 (1.07-1.46)*	65.2 (911)	1.27 (1.12-1.45)*
Others[&]	43.6 (283)	1.31 (0.95-1.81)***	57.8 (370)	1.38 (1.13-1.69)*
**Caste[^]**				

General	49.9 (1,491)	1.00	56.6 (1,688)	1.00
Other Backward Class	53.6 (2,242)	0.90 (0.78-1.03)	58.5 (2,464)	1.02 (0.91-1.14)
Scheduled Castes	56.9 (1,223)	0.93 (0.78-1.11)	63.6 (1,376)	1.02 (0.89-1.16)
Scheduled Tribes	40.9 (353)	0.59 (0.46-0.74)*	43.7 (382)	0.68 (0.56-0.83)*
**Place of residence**				
Rural	57.1 (3,834)	1.00	62.4 (4,236)	1.00
Urban	42.3 (1,478)	0.94 (0.83-1.07)	48.6 (1,681)	1.01 (0.91-1.11)
**Total**	52.0 (5,312)	--	57.7 (5,917)	--

*****significant at p<0.05; **not significant at p<0.05 but significant at p<0.1; #Includes widow, separated and divorced; +0 years of schooling also includes those who never attended school; ++includes cigarette and *biddi* both and presently smoking or ever smoked were categorised as yes and never as no; +++presently using and ever used categorised as yes and never as no;; &includes Sikh, Christian, Jain, Buddhism and others; ^Caste system is a sort of social class system in which people are classified based on culture and occupation [13], $Clean only includes LPG and Others includes firewood, crop residual, cow dung cake, coal and kerosene which were used for any purpose
